# Toxicological Impacts of Polypropylene Nanoparticles Similar in Size to Nanoplastics in Plastic-Bottle Injections on Human Umbilical Vein Endothelial Cells

**DOI:** 10.3390/toxics13090802

**Published:** 2025-09-21

**Authors:** Jie Wang, Zhong-Lan Chen, Cheng-Gang Liang, Hui-Ying Yang, Xian-Fu Wu, Hui-Min Sun

**Affiliations:** Standard Substances and Standardization Management Center, National Institutes for Food and Drug Control, Beijing 100260, China; sunhm_lab@163.com (J.W.);

**Keywords:** injections, polypropylene nanoparticles, inflammatory response, apoptosis, cell damage

## Abstract

Microplastic and nanoplastic (MNP) particles have been observed in various human organs. However, polypropylene (PP), one of the top three most commonly detected types of MNPs in terms of quantity, is also present in injections given for the infusion treatment of diseases, and there is a considerable knowledge gap concerning its adverse effects on the human cardiovascular system. In this study, we used commercial PP particles (500 nm), similar in size to nanoplastics (NPs) present in injections and greater than or equal in concentration to NPs in the blood of healthy individuals, as the experimental dose to study their toxicological effects on human umbilical vein endothelial cells. The results revealed that PP particles at 35 μg/mL, equivalent to 20 times the concentration of blood, reduced cell viability, induced oxidative stress, caused cytomembrane damage, increased the inflammatory response, promoted apoptosis, and inhibited cell migration and wound tissue healing. In addition, a NP concentration of up to 210 μg/mL decreased the level of zonula occludens-1. In conclusion, since we used spherical particles, a type of nanoplastic present in plastic-bottle injections in clinical treatment that induces toxicological effects, this study provides cellular-level insights into the ecological risks of NP exposure in the human body.

## 1. Introduction

Plastic products are organic polymer materials that have gradually been further introduced into peoples’ lives in various forms, such as packaging materials for various drinks, food, and drugs; skin care products; and excipients added to skin care products. Although they are convenient for everyone, they also introduce potential risks. If these plastic bottles are not properly disposed of after use, they will be broken or degraded through physical, chemical, or biological external effects, producing many microplastic (MP, *Φ* < 5 mm) and nanoplastic (NP, *Φ* < 1 μm) particles. These MNP particles can indirectly enter essential goods through the circulation of water, soil, and the atmosphere [1,2,3]. At present, MNPs have been detected in meat, vegetables, fruits, grain, water, beverages, salt, personal skin care products, injections, and household items, and polypropylene is one of the top three most commonly identified types [4,5].

**Figure 1:**
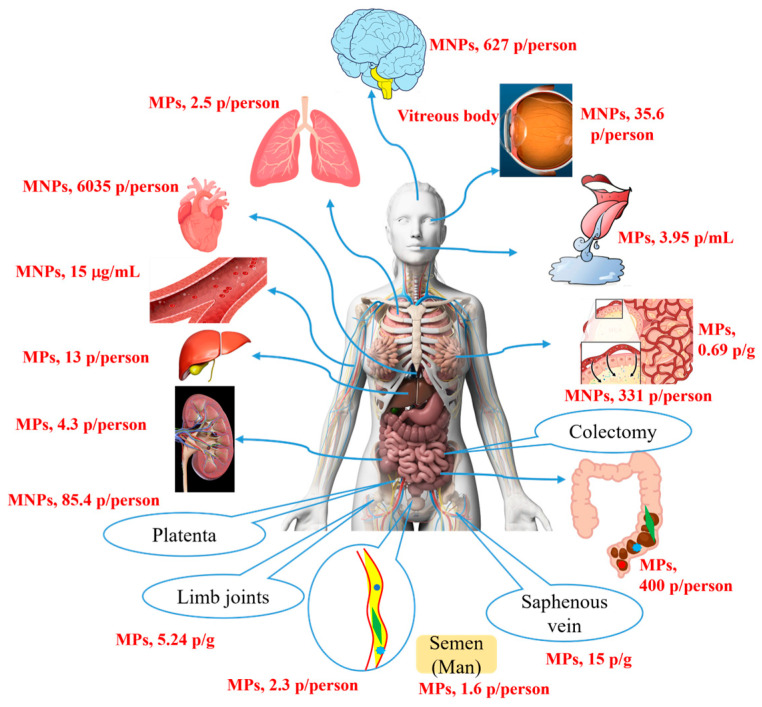
The accumulation and excretion of micro- and nanoplastics in the human body.

MNP particles can potentially enter various human tissues and organs directly—through ingestion, breathing, and skin injury—or indirectly—through entering blood circulation. Some are accumulated in tissues, and the rest are excreted. MPs of 0.8–1.6 μm and 1.6–5.6 μm in size have been detected in human colectomy specimens and the lungs of nonsmoking patients, respectively [6,7]. Polystyrene nanoplastics (PS-NPs) (≤240 nm) can penetrate the air-blood barrier and enter the lungs and blood vessels via inhalation through the nose [8,9]. NPs smaller than 100 nm can be used as carriers of polybrominated diphenyl ethers, harmful substances that can be carried into the human blood through damaged skin [10]. MNP particles entering the blood can accumulate in the liver, heart, saphenous vein, limb joints, lung, reproductive organs, placentae, and kidney organs through the circulatory system [7,11,12,13,14,15,16,17], and NP particles smaller than 200 nm can even cross the blood–brain barrier (BBB) and accumulate in the brain and eyeball [18,19,20]. Nevertheless, although ingested MP particles can be excreted in large quantities (20–800 μm) from feces [21,22], only trace amounts of MP particles entering the body through other pathways can be secreted via body fluids. PP-MPs can be secreted into breast milk (2–12 μm), human urine (4–15 μm), and semen (2–6 μm) [23,24,25]. Statistical findings reveal that the cumulative amount within tissues and organs is far greater than the excreted amount (Figure 1), particularly in the heart and brain tissues [12,17]. Moreover, a study tracking investigations into MNP accumulation showed that accumulation in the brain and liver in 2024 was significantly higher than that in 2016. Radiation imaging experiments revealed that PS-MNPs (1 μm, 20 nm) primarily accumulated in the organs of mice 168 h after intravenous injection, with ratios of 80% and 30%, respectively, while only 15% and 52% were excreted in the urine [26]. Similarly, after intravenous injection for 4 h, PS-NPs (100 nm, 80 μg) accumulated in the liver, spleen, and lungs of the mice, and no significant excretion was detected at 28 days [27].

When the accumulated concentration is lower than the environmental concentration, MNP particles also have a certain degree of toxicity to normal tissue cells and organoids (Table 1), creating a potential risk to human health. In particular, PS-MNPs at a concentration of 5 μg/mL, lower than that found in water (40 μg/mL), promoted the carcinogenesis of normal colon fibroblasts (CCD-18Co) [28], which may be a promoting factor for rectal cancer in individuals working in the plastics and rubber industries [29]. PS-MNPs can cause metabolic disorders in the normal human hepatocytes L02 [30], and reduce glucose metabolism and antioxidative stress in HEK293 cells [31]. Polyethylene terephthalate microplastics (PET-MPs) (10 μg/mL) detected in bottled drinking water can inhibit proliferation and decrease the differentiation potential of marrow mesenchymal stromal cells (BMMSCs) and adipose mesenchymal stromal cells (AMSCs) [32]. Moreover, PS-MNPs have been shown to induce neurotoxicity in forebrain cortex spheroids [33], apoptosis and inflammation in intestinal organoids [34], and metabolic dysfunction and lipid accumulation in H1-derived hepatoid organs [35]. Surprisingly, compared to typical pregnant women, the presence of polyethylene (PE) and PS (2–38 p/person) MPs in the placentas of other women is negatively correlated with newborn height, and weight [36]. Unlike MPs, NPs can enter cells and cross the BBB, and they may have more toxic effects, such as inducing dementia [19]. Moreover, 500 nm PS particles can only induce cytomembrane damage, whereas the 100 nm particles can be absorbed into the cytoplasm, as well as induce autophagosome formation and autophagic flux blockage [37]. Polyamide nanoplastics (PA-NPs) that cross the BBB may cause neurotoxicity and vitreous opacification of the eyeball [19,20]. Unfortunately, there are few studies on the toxicological effects of PP-NPs on normal human cells and organoids.

Both MNPs themselves and the additives contained within them may cause toxicological damage to cells. Endocrine-disrupting chemicals (EDCs) are natural or synthetic substances that can interfere with hormonal systems and alter their physiological signaling [38]. EDCs may trigger the development of hormone-sensitive cancers by promoting cell proliferation and increasing the number of random genetic errors, such as prostate cancer, breast cancer, ovarian cancer, testicular germ cell cancer, etc. [39]. Moreover, the signaling hub protein RACK1 (Receptor for Activated C Kinase 1), a relevant EDC target that responds to steroid-active compounds, could be considered a molecular bridge between the endocrine-regulated tumor microenvironment and the innate immune system [40]. At the early stage, as we have acknowledged, MNPs contain EDCs such as bisphenol A, BPS, and 1,3,5-trimethyl-2,4,6-tris(3,5-di-tert-butyl-4-hydroxybenzyl)benzene [41], and BPA can directly (through glucocorticoid receptor α agonism) and indirectly (through an increased glucocorticoids release due to HPA axis hyperactivation) induce RACK1 downregulation, leading to an anxiety-like behavior and neuroinflammation [38], while BPS acts predominantly through androgen receptor (AR)-RACK1 pathway, as well as through the G protein-coupled estrogen receptor (GPER)-AR-RACK1 to a lesser extent [42]. Some studies have regarded MNPs as carriers of EDCs, which can interfere with the effects of different hormone signaling pathways and induce functional disorders in the gonads, thyroid, and adrenal glands through the HP axis [43]. NPs (<100 nm) and plastic additive co-exposure modulate estrogen–androgen–thyroid–steroidogene (EATS) pathways and can disrupt fish embryo–larval development in the F1 generation [44]. Nevertheless, the latest research indicates that MNPs themselves are also regarded as EDCs, and they have harmful effects on the human reproductive systems [45]. When PS-NPs overload the lysosomes of human placental primary trophoblast cells, they induce autophagy and activate the Nrf2/HO-1 antioxidant pathway, ultimately leading to endocrine disruption through decreasing in ß-hCG levels in the extracellular compartment [46]. PS-NPs can increase the expression of the SOD1 gene and decrease the level of estrogen triol in H295R cells [47]. PS-MPs can reduce the testosterone level in mice through Glutathione peroxidase 1 (GPX1)–PERK–eukaryotic translation initiation factor 2α (EIF2α)–Activating Transcription Factor 4 (ATF4)–C/EBP homology protein (CHOP)–SRD5A2 signaling pathways, as well as disrupt the testosterone balance through the HPG axis [48]. PS-MPs can induce a significant increase in luteinizing hormone, simultaneously causing significant decreases in estradiol and follicle-stimulating hormone levels; the hormonal imbalance eventually leads to polycystic ovary syndrome and ovarian fibrosis [49].

Owing to NPs’ potential accumulation in the human body after entering tissues, PP materials account for a high proportion of MNP types and are commonly present in PP-bottle injections given for the infusion treatment of diseases [4]. After entering the blood through intravenous injection, PP-NPs reach the rest of the body through the circulatory system, and first contact occurs with vascular endothelial cells. Therefore, it is urgent to confirm whether PP-NP particles have toxic effects on human vascular endothelial cells. In fact, NPs in the plastic-bottled injections used for clinical infusion therapy are irregular in shape and difficult to transfer. In this study, spherical PP particles (*Φ* = 500 nm) similar in size to those that appeared in the injections were selected, and the cumulative infusion volume (10.9 bottles) for Chinese people per year was used as the experimental dose for treating human umbilical vein endothelial cells. The toxicological effects of PP particles on those cells were studied by detecting changes in inflammatory factors, apoptosis, cell proliferation, and antioxidant stress. The adverse effects of polypropylene nanoplastic particles in PP-bottle injections on cardiovascular system cells were first studied.

## 2. Materials and Methods

### 2.1. Materials

Human umbilical vein endothelial cells (#PCS-100-013), vascular cell base medium (#PCS-100-030), and endothelial cell growth kit (#PCS-100-041) were purchased from the American Type Culture Collection (ATCC, Rockefeller, MD, USA). Penicillin Streptomycin (#15140-122) was purchased from Gibco (Grand Island, CA, USA). LPS (Lipopolysaccharide from *Escherichia coli* O55:B5, #L6529) and intracellular reactive oxygen species (ROS) detection kit (#96992) were purchased from Sigma-Aldrich (St. Louis, MI, USA). Lactate dehydrogenase (LDH) activity assay (#ab102526), recombinant anti-LC3B antibody [EPR18709]-autophagosome marker (#ab192890), and goat anti-rabbit IgG H&L (Alexa Fluor^®^ 488, # ab150077) were purchased from Abcam (Boston, MA, USA). Human IL-2 (#430207), IL-6 (#431807), IL-10 (#430507), and TNF-α (#430607) enzyme-linked immunosorbent assay (ELISA) kits were purchased from BioLegend (San Diego, CA, USA). HiPure Total RNA Plus Mini Kit (#R4121-02) was obtained from Magen (Guangzhou, China). The PrimeScript^TM^ FAST RT reagent Kit with gDNA Eraser (#RR092A) and TB Green^®^ Premix Ex Taq^TM^ II FAST qPCR (#CN830A) were obtained from Takara (Dalian, China). MicroAmp Optical 8-Tube Strips (#4316567) and MicroAmp Optical 8-Cap Strips (#4323032) were purchased from Applied Biosystems (Carlsbad, CA, USA). Transwell^®^ cell culture chambers (#3422) and cell culture plates (#3513) were purchased from Corning (Corning, NY, USA). Annexin V-fluorescein isothiocyanate (FITC)/Prodium Iodide (PI) cell apoptosis detection kit (#abs50001) was obtained from Absin (Shanghai, China). Confocal dishes (Coverglass Bottom Dish, #YA0571), 0.1% crystal violet ammonium oxalate solution (#G1063), and 4% paraformaldehyde (#P1110) were purchased from Solarbio Life Sciences (Beijing, China). Polypropylene nanoparticles (#PP1370D) were purchased from Hubei Xinyuhong Biotechnology Co., Ltd. (Wuhan, China). Tween 80 (#30189828) was purchased from Sinopharm Chemical Reagent Co., Ltd. (Shanghai, China).

### 2.2. Cell Culture and Preparation of PP-NPs

Since NP particles present in PP-bottle injections enriched on filter membranes cannot be transferred efficiently, we chose commercial PP nanoparticles identical in size and investigated their toxicological effects on human umbilical vein endothelial cells.

HUVECs were cultured in vascular cell basal medium supplemented with an endothelial cell growth kit for VEGF at 37 °C with 5% CO_2_. The 5th to 10th generations of cells were used for assays. The culture flasks and wells purchased all underwent TC treatment. A 10.5 mg/mL suspension of PP particles was prepared in culture medium, followed by adding Tween 80 to 0.01% *v*/*v*. The mixture was ultrasonicated at 40 kHz for 1 h with an ultrasonic cleaner (TYD-B120, Beijing Taiyuandachuang Industrial Equipment Co., Ltd., Beijing, China). Based on the reported concentrations of NPs (1.6 μg/mL) in the blood of 22 anonymized, healthy non-fasting adults (the gender ratio and previous medical histories were unknown) [50], additional experimental concentrations were prepared, as detailed above. The suspension was ultrasonicated for 0.5 h before the cells were treated. The cells were treated with a culture medium containing 0.01% Tween 80, with this formulation serving as the negative control group. Finally, after mixing with water (0.01% Tween 80), the PP particles were enriched onto the Al_2_O_3_ membrane through filtration, and then they were observed using a scanning electron microscope.

### 2.3. Cell Viability

Cell viability was measured using a CCK-8 assay. Firstly, HUVECs (5 × 10^3^ cells/well) were planted in 96-well plates and treated with concentrations (0, 1.5, 17.5, 35, and 210 μg/mL) of 0.5 μm PP-NPs for 48 h. Subsequently, the cells were incubated for 3 h after adding the CCK-8 reagent. Absorbance was measured at 450 nm via a microplate reader (PerkinElmer Instruments Inc., Waltham, MA, USA). Triton X-100 (5%) was used as a positive control to treat the cells in the same manner as described above.

### 2.4. Detection of Reactive Oxygen Species (ROS)

Intracellular ROS (superoxide and hydroxyl radicals) levels were measured through a fluorometric intracellular ROS kit (MAK143, Sigma-Aldrich, Madrid, Spain). This kit provides a fluorogenic sensor that reacts with ROS, providing a fluorometric product proportional to the amount of ROS present in HUVECs. To perform these experiments, the cells were seeded into 96-well black plates with clear bottoms for fluorometric assays (20,000 cells per well) in medium for 48 h in the presence or absence of PP-NPs. A volume of 100 μL of Master Reaction Mix containing the fluorogenic sensor was subsequently added per well, and the mixture was incubated in a 5% CO_2_, 37 °C incubator for 1 h. H_2_O_2_ (1 mM) was used as a positive control, and the fluorescence intensity was measured at λ_ex_ = 490/λ_em_ = 525 nm via a microplate reader (PerkinElmer Instruments Inc., Wellesley, MA, USA).

### 2.5. Lactate Dehydrogenase (LDH) Activity Assay

Damage to the cell membranes caused by PP-NPs was measured via an LDH activity assay. In brief, the cells were treated with different concentrations (0, 1.5, 17.5, 35, and 210 μg/mL) of 500 nm PP-NPs for 48 h. The cell culture supernatants were centrifuged (10,000× *g*, 10 min, 4 °C) and collected. According to the manufacturer’s instructions, an LDH substrate mixture was subsequently added to the diluted cell culture supernatants, and the optical density (OD) values were measured via a microplate reader (PerkinElmer Instruments Inc., Wellesley, MA, USA) at 450 nm in kinetic mode for 60 min. LDH activity was normalized to the corresponding protein content.

### 2.6. Cytokine Test

HUVECs (ATCC, USA) were used to observe cytokine release after PP-NP treatment (0, 1.5, 17.5 and 35 μg/mL, respectively). Lipopolysaccharide (LPS) and proinflammatory cytokines such as TNF-α initiated an immune response. The levels of cytokines produced by HUVECs treated with PP particles could potentially indicate an active immune response to PP particles in human blood vessels. To confirm this, HUVECs were seeded at a density of 2 × 10^4^ cells/well in 96-well plates. PP-NPs were added, and the plate was incubated in a 5% CO_2_, 37 °C incubator for 3 days; moreover, 10 μg/mL of LPS was used as the positive control. The cell culture supernatants were centrifuged (10,000× *g*, 10 min, 4 °C) and collected for ELISA (IL-2, IL-6, IL-10, and TNF-α). ELISA was performed according to the manufacturer’s instructions.

### 2.7. Wound Healing Assay

HUVECs (1 × 10^5^ cells/well) were seeded in a 12-well plate and grown to a 100% density; then, a pipette tip was used to make a scratch on the cellular layer. Different doses of PP-NPs (0, 1.5, 17.5, 35, and 210 μg/mL) were added into the wells, and the cells were continuously cultured in 5% CO_2_, 37 °C incubator for 24 h. Subsequently, 4% paraformaldehyde (PFA) solution (Solarbio, Beijing, P1110) and 0.1% crystal violet (Solarbio, Beijing, G1063) were used to fix and stain the cells for 10 min sequentially. After washing, a microscope (Nikon TS2-FL, Yokohama, Japan) was used to capture the images, and the wound area was calculated via ImageJ software (1.8.0, NIH, Bethesda, MD, USA).

### 2.8. Transwell Migration

HUVECs (1 × 10^4^ cells) were seeded into Transwell inserts containing 100 μL of medium with or without PP-NPs (0, 1.5, 17.5, 35, 210, and 350 μg/mL), and 500 μL of complete medium was added into the corresponding lower wells. Next, 4% PFA was used to fix the cells after 24 h of incubation. Then, a cotton swab was used to remove the remaining cells on the upper side of the Transwell membrane, and 0.1% crystal violet was used to stain the migrated cells on the lower side for 10 min. Finally, images were captured using a microscope (Nikon TS2-FL, Yokohama, Japan), and ImageJ software (1.8.0, NIH, Bethesda, MD, USA) was used to count the migrated cells.

### 2.9. Annexin V-FITC and PI Staining

Cell apoptosis was studied via an Annexin V-FITC/PI Apoptosis Detection Kit (#abs50001, Absin, China). The cells were incubated with or without PP-NPs of different doses (1.5, 17.5, 35, 1050 μg/mL) for 48 h. Subsequently, cells were collected after trypsinization and washed twice with PBS. Then, cells were resuspended in 1× binding buffer and stained with 5 μL of Annexin V-FITC for 15 min. Finally, the cells were stained with PI for 5 min before we analyzed the apoptotic/necrotic cell population via the LSRFortessa™ system (BD Biosciences, San Jose, CA, USA).

### 2.10. RNA Extraction and Real-Time PCR

Total RNAs extracted from HUVECs (1 × 10^6^ cells/group) treated with PP-NPs (0, 1.5, 17.5, 35, and 210 μg/mL, respectively) for 48 h were extracted via the HiPure Total RNA Plus Mini Kit and reverse-transcribed via the PrimeScript^TM^ FAST RT Reagent Kit. Then, real-time PCR was performed with a TB Green^®^ Premix Ex Taq^TM^ II FAST qPCR Kit (Takara) using QuantStudio 5 (ABI, Des Moines, IA, USA). The mRNA levels of interleukin 2, 6 (*IL2*, *6*), tumor necrosis factor α (*TNF-α*), zonula occludens-1 (*ZO-1*), *caspase-3*, tumor protein 53 (*p53*), B-cell lymphoma-2 (*Bcl-2*), Bcl-2 associated X protein (*Bax*), the NLR family, and pyrin domain-containing protein 3 (*NLRP3*) were normalized to *GAPDH* and *β-actin*, respectively (Table 2).

### 2.11. Microtubule-Associated Protein 1 Light Chain 3 (LC3) Test (Immunofluorescence Staining)

After HUVECs were incubated with PP-NPs (300 and 500 nm) for 48 h, they were washed twice with PBS and fixed with 4% PFA/PBS for 10 min. After the permeabilization of fixed cells with 0.2% Triton X-100/PBS for 15 min, block incubation with 3% bovine serum albumin dissolved in PBS was performed for 60 min. Subsequently, HUVECs were incubated with an LC3 antibody (1:50) at 4 °C overnight and then treated with a secondary antibody (1:50) at room temperature for 30 min. After nuclear staining with DAPI for 10 min, the cells were washed thrice and ultimately imaged under a fluorescence microscope (Zeiss LSM 900, Jena, Germany). The LC3-expressed area was calculated via ImageJ (NIH, Bethesda, MD, USA).

### 2.12. Statistical Analysis

The results are represented as the means ± SEM of five independent experiments. Statistical differences were evaluated via Student’s t-test and Tukey’s test, with differences considered significant at *p* < 0.05, as indicated in the figure legends. Statistical evaluation was performed using SPSS 25.0 (Chicago, IL, USA), and graphs were prepared using GraphPad Prism 5 (San Diego, CA, USA).

## 3. Results

### 3.1. PP-NPs Reduce the Viability of HUVECs in a Dose-Dependent Manner

**Figure 2:**
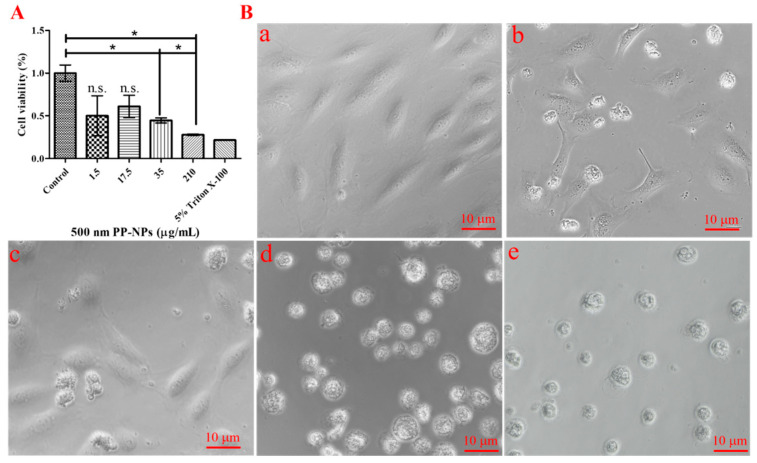
The adverse effect of PP-NPs with 500 nm on cell viability. (**A**) Changes in cell viability caused by PP-NPs. HUVECs were exposed to serial concentrations 0 (**a**), 1.5 (**b**), 17.5 (**c**), 35 (**d**), and 210 (**e**) μg/mL of 500 nm PP-NPs (**B**) for 48 h, respectively. The cell viability was measured by the CCK-8 assay. Results were presented as mean ± SEM (*n* = 5). n.s.: no significance. * *p* < 0.05.

To verify whether PP-NPs affect the viability of HUVECs, we treated them with different concentrations (1.5, 17.5, 35, and 210 μg/mL) of 0.5 μm PP-NPs. The most significant decrease in viability appeared in HUVECs incubated with higher concentrations of 0.5 μm PP-NPs for 48 h (Figure 2). The respective inhibitory rates of the cells treated with 35 and 210 μg/mL of PP-NPs after 48 h were 55% and 72%, but no significant inhibition was observed in the cells treated with 1.5 and 17.5 μg/mL (Figure 2A). Moreover, the state of the HUVECs’ treated with 0.5 μm PP-NPs is exhibited in Figure 2B. In summary, the dose-dependent inhibition of endothelial cell viability might be related to the interaction between PP-NPs and cytomembranes.

### 3.2. Necrosis, Not Autophagy, Mediated PP-NP-Induced Decrease in Viability

**Figure 3:**
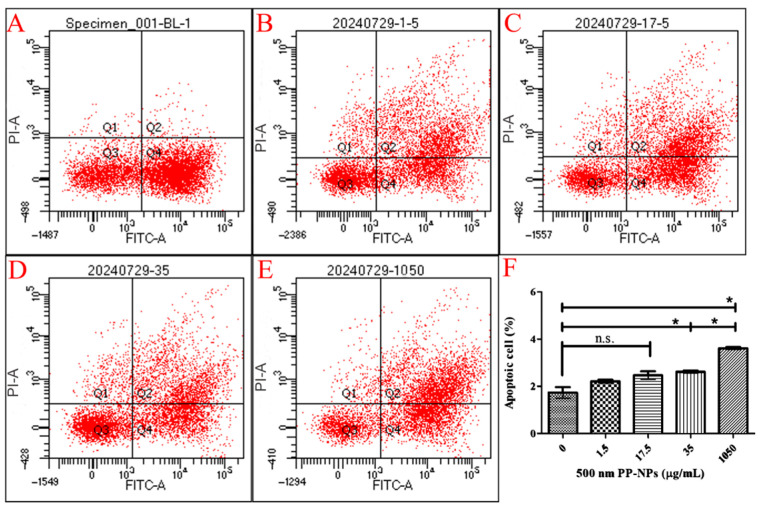
The effect of PP-NPs on necrosis of HUVECs. HUVECs were exposed to 500 nm PP-NPs for 48 h. Cells were digested by trypsin and stained with Annexin V-FITC and PI. Apoptotic/necrotic cell populations were analysed by flow cytometry. (**A**–**E**) Dot plot. (**F**) Apoptosis cell rates were calculated. Data are expressed as mean ± SEM (*n* = 5). n.s.: no significance, * *p* < 0.05.

To elucidate whether this decreased viability is associated with cell apoptosis, flow cytometry was performed on PP-NP-treated HUVECs (Figure 3). Annexin-V and PI staining revealed an increase in necrotic cell death, especially at concentrations of 35 μg/mL (early/late apoptotic cells: 20.1%/21.2%; necrotic cells: 5.2%) and 1050 μg/mL, which induced significant increases in late cell apoptosis (Figure 3F). However, both 300 and 500 nm PP particles barely induced the accumulation of autophagosomes in HUVECs (Figures S1 and S2). These results confirmed that apoptosis and necrosis contributed to decreased cell viability without changes in the proportions measured during early and late apoptosis.

### 3.3. PP-NPs Induced Oxidative Stress and Cell Membrane Damage in HUVECs

**Figure 4:**
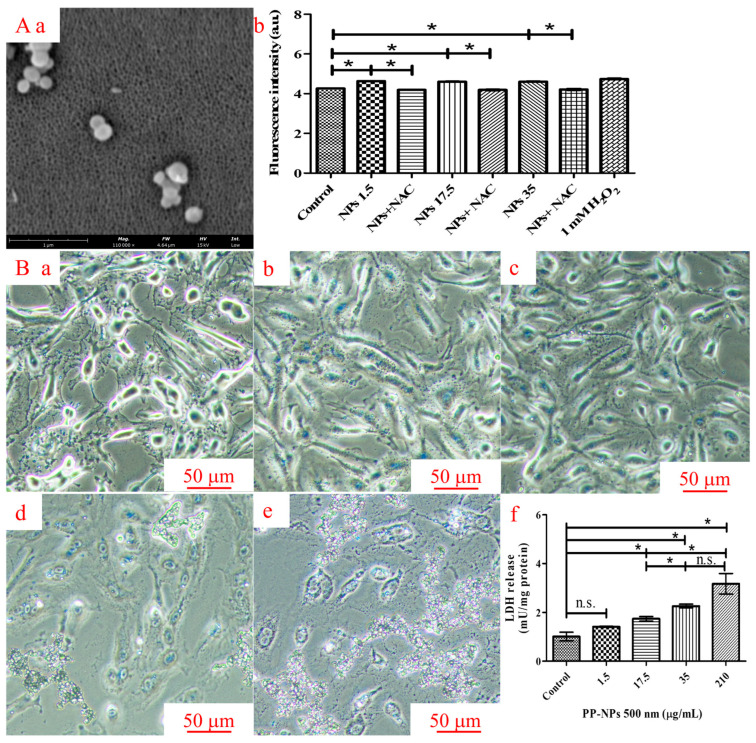
500 nm PP-NPs induced oxidative stress and cytomembrane damage. (**Aa**) SEM images of PP particles (scale bar = 1 µm; magnification, ×110,000). (**Ab**) Relative ROS quantitative results for fluorescence intensity. For (**Ab**,**Ba**–**Be**), cells were exposed to serial PP-NP concentrations (0, 1.5, 17.5, and 35 μg/mL) for 48 h (scale bar = 50 µm). (**Bf**) The levels of LDH in cell supernatants after exposure to PP-NPs. NAC, N-Acetylcysteine (5 mM). Representative graphs were selected, and the data were the mean ± SEM of 5 independent experiments. n.s.: no significance, * *p* < 0.05.

To study the other causes of reduced cell viability, we also evaluated oxidative stress and cell membrane damage in HUVECs exposed to serial concentrations of 500 nm PP-NPs for 48 h (Figure 4). As shown in Figure 4Aa,Ab, compared with the control groups, 500 nm PP-NPs (1.5, 17.5, 35, and 210 μg/mL) induced significant increases in intracellular ROS generation. To further study the distribution of PP-NPs in HUVECs, a fluorescence microscope was used to determine whether PP-NPs adhered to the cell membrane. Micrographs revealed that 500 nm PP-NPs adhered to the surface of the cell membranes after 48 h of exposure (Figure 4Bb–Be). LDH activity is an important indicator of cell membrane damage. As shown in Figure 4Bf, LDH activity in the cell culture supernatants significantly increased in response to treatment with 500 nm PP-NPs at concentrations of 17.5, 35, and 210 μg/mL for 48 h. In summary, 500 nm PP-NPs interacted with the cell membrane, damaging it.

### 3.4. PP-NPs Inhibit the Migration of HUVECs

**Figure 5:**
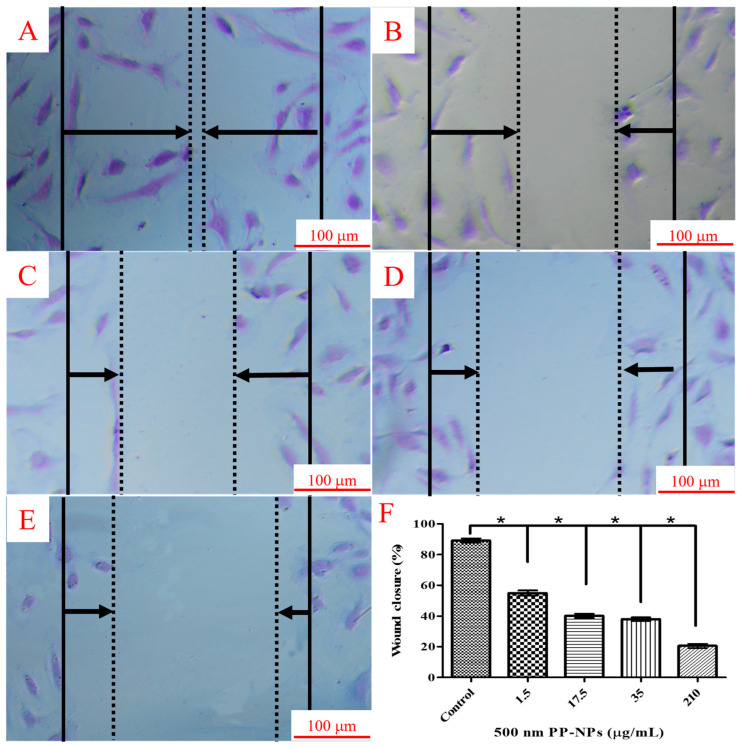
PP-NPs inhibited the wound closure of HUVECs. (**A**–**E**) HUVECs (1 × 10^5^ cells/well) were seeded in a 12-well culture plate. After achieving 100% confluence, a pipette tip was used to make a scratch and the cells were exposed to 500 nm PP-NPs at different concentrations (0, 1.5, 17.5, 35, and 210 μg/mL) for 24 h. Cells were fixed with 4%PFA and stained using 0.1% crystal violet. (**F**) The wound area was calculated with ImageJ. Values are presented as mean ± SEM (*n* = 5). Significance is indicated as * *p* < 0.05. (0.01% *v*/*v* Tween 80/medium)-treated control group (wound closure is close to 100%). Scale bar: 100 μm. Solid line: the boundaries of the cells that were removed earlier; arrows: the direction of cell extension; dashed line: the new boundaries formed after cell growth.

**Figure 6:**
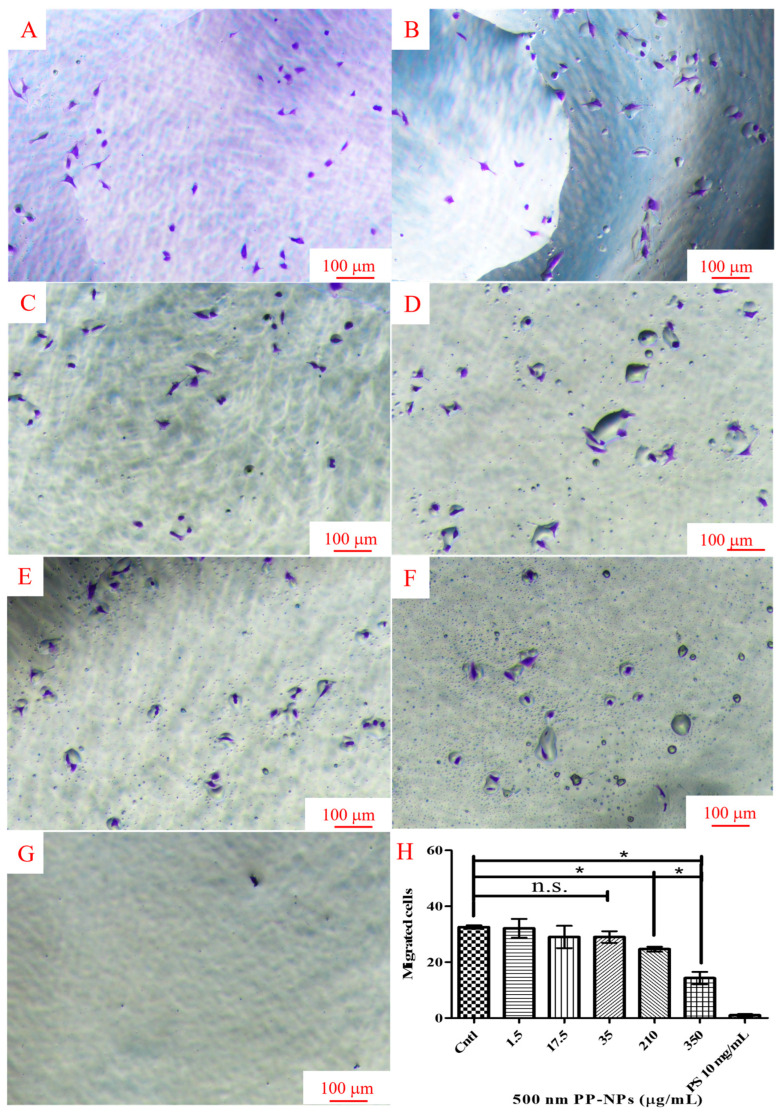
PP-NPs inhibited the migration of HUVECs. (**A**–**G**) Cells (1 × 10^4^ cells/well) were seeded into a Transwell system in 100 μL of medium containing 500 nm PP-NPs; then, 500 μL of medium without PP-NPs was added into the lower chamber. After incubation, cells on the Transwell membrane were removed, and the migrated cells under the membrane were fixed and stained. (**H**) ImageJ was used to count the stained cells. Data are presented as the mean ± SEM (*n* = 5). Cntl, control. Significance is indicated as * *p* < 0.05. n.s.: no significance, Scale bar = 100 μm.

Wound healing and Transwell migration experiments were performed in HUVECs treated with 1.5, 17.5, 35, and 210 μg/mL of 0.5 μm PP-NPs to verify whether they affects the tube-forming capacity. Primarily, the wound healing assay exhibited 90% wound closure in the control group but 54%, 41%, 39%, and 21% wound closure in cells treated with 1.5, 17.5, 35, and 210 μg/mL of 0.5 μm PP-NPs (Figure 5). Similarly, the Transwell experiment indicated that the chemotactic migration of HUVECs was significantly reduced by treatment with 0.5 μm PP-NPs, with reductions of 23% and 57% at 210 and 350 μg/mL, respectively (Figure 6). These data reveal that the inhibition of the migratory activity of HUVECs by PP-NPs may provide insights into angiogenesis inhibition.

### 3.5. PP-NP-Induced Inflammation

**Figure 7:**
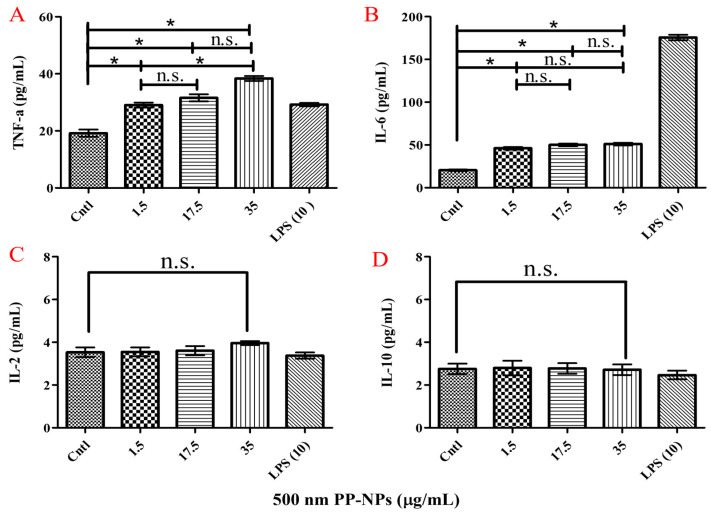
500 nm PP-NPs induced inflammatory responses in HUVECs. (**A**–**D**) The protein levels of *TNF-α* (**A**), *IL6* (**B**), *IL2* (**C**) and *IL-10* (**D**). For (**A**–**D**), cells were incubated with serial concentrations (0, 1.5, 17.5 and 35 μg/mL) of 500 nm PP-NPs for 72 h, respectively. The data were mean ± SEM (*n* = 5). * *p* < 0.05. LPS, 10 μg/mL. n.s.: no significance.

To determine whether any immune response was triggered in cells treated with PP particles, we evaluated the inflammatory response in HUVECs incubated with different concentrations of 500 nm PP-NPs for 72 h (Figure 7). In this study, the protein expression of inflammatory cytokines (*IL-6* and *TNF-α*) increased significantly following the 72 h exposure of HUVECs to 500-nm PP-NPs at concentrations of 1.5, 17.5, and 35 μg/mL (Figure 7A,B). However, there were no obvious differences in *IL-2* and *IL-10* levels in HUVECs treated with 500 nm PP-NPs, as mentioned above (Figure 7C,D). These data indicated that inflammation-related cytotoxicity induced by 500 nm PP-NPs occurred in HUVECs.

### 3.6. PP-NPs Induce Changes in Cytokines in HUVECs

**Figure 8:**
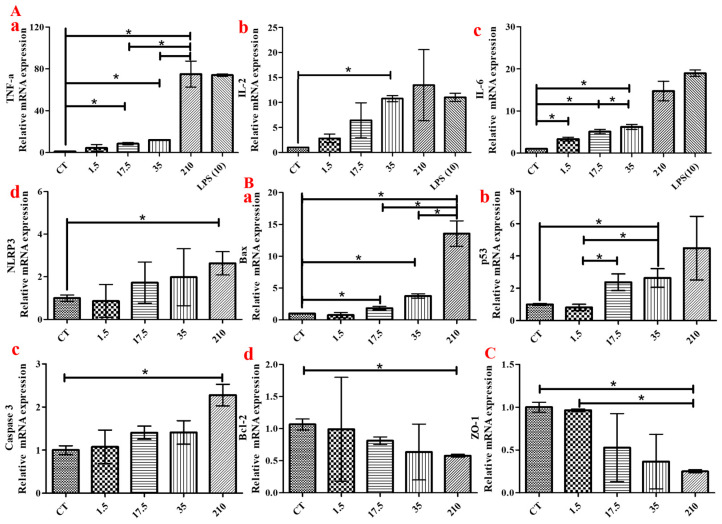
500 nm PP-NPs induced inflammatory responses, apoptosis, and decreased in intercellular junction function in HUVECs. (**Aa**–**Ad**,**Ba**–**Bd**,**C**) The mRNA levels of *TNF-α* (**Aa**), *IL2* (**Ab**), *IL6* (**Ac**), *NLRP3* (**Ad**), *Bax* (**Ba**), *p53* (**Bb**), *Caspase-3* (**Bc**), *Bcl-2* (**Bd**) and *ZO-1* (**C**). Cells were incubated with different concentrations (0, 1.5, 17.5, 35 and 210 μg/mL) of PP-NPs for 48 h. The data were mean ± SEM (*n* = 5). The mRNA levels of the above factors were normalized to *GAPDH*. * *p* < 0.05.

To further verify the possible inflammatory response, cell apoptosis, and vascular barrier damage caused by PP-NPs, we finally evaluated cytokines at the mRNA level in HUVECs exposed to serial concentrations of 500 nm PP-NPs for 48 h (Figure 8). The concentrations of inflammatory cytokines *TNF-α* and *IL-6* increased in a dose-dependent manner as the PP-NPs concentration increased (Figure 8Aa,Ac), and significant *IL-2* and *NLRP3* level increases were induced by 500 nm PP-NPs at concentrations of 35 and 210 μg/mL (Figure 8Ab,Ad). In addition, a significant dose-dependent increase in the proapoptotic factor *Bax* was induced by increasing the PP-NP concentration (Figure 8Ba), and PP-NP concentrations of 35 and 210 μg/mL also promoted significant increases in *p53* and *caspase 3*, respectively (Figure 8Bb,Bc) but inhibited the production of the antiapoptotic factor *Bcl-2* (Figure 8Bd). Furthermore, the production of ZO-1 was significantly inhibited by 210 μg/mL PP-NPs (Figure 8C). The data normalized against the levels of GAPDH mRNA were similar to those for β-actin (Figure S3). These results suggested that inflammation-, apoptosis-, and intercellular junction function disruption-related cytotoxicity were induced by 500 nm PP-NPs in HUVECs.

## 4. Discussion

All the data are normally distributed (Figure S4). Studies conducted in mammals have shown that NPs, especially those derived from PP plastic-bottle injections, can enter the blood circulation directly and be transported to remote organs. However, until now, little information has been available regarding the potential risks posed by PP-NPs to the cardiovascular system. In this study, we used HUVECs as a vascular endothelial cell model to investigate the influence of 500 nm PP-NPs on the cytotoxicity and further analyzed the differential autophagy effects of PP-NPs on these cells. In the experimental results, we found that 35 μg/mL NP particles, equivalent to 20 times the concentration of nanoplastics in the blood of healthy individuals, could induce oxidative stress, reduce cell viability and junction function, cause cell membrane damage, increase the levels of the inflammatory factors *TNF-α* and *IL-6*, increase the levels of the apoptotic factors *Bax* and *caspase-3* to induce cell apoptosis, and inhibit cell migration and wound tissue healing.

When they have the same diameter, due to the possible existence of concave areas in the irregularly shaped nanoplastics, their surface area is larger than that of the spherical PP particles [51]. The zeta potential of the filtrate after the injection solution was filtered through a GF-B membrane (*Φ* = 1 μm) was −3.41 mV, which was the potential of the remaining nanoplastic particles (Table S1; Figure S5). The spherical PP particles used in this study had a surface area of 0.283 μm^2^ and a zeta potential of −3.15 mV (the injection solution dispersed with the 500 nm PP particles after filtration through an Al_2_O_3_ membrane with a pore size of 20 nm). We speculated that the NPs in the injections may have been irregularly shaped or that there may have been a mixture of irregularly shaped and spherical NPs. The larger the absolute value of the charge, the more stable the particle system [52], so the toxicity to cells caused by NPs present in injections may be greater than that of PP particles. In summary, PP particles can indirectly reflect the toxic effect of nanoplastics in injections on human umbilical vein endothelial cells.

Regarding the clinical relevance of PP-NP doses used in this study, we estimated that an average of 24 μg PP-NPs per year are administered intravenously to humans [4]. Based on the concentration of nanoplastics in the blood of healthy individuals, we designed experimental doses approximately 1, 10, and 20 times the normal level to conduct toxicological studies on HUVECs. To determine the number of cumulative years that could cause pathological harm, a maximum dose of 1050 μg/mL was chosen.

In this study, we initially found that 500 nm PP-NPs interacted with HUVECs in a concentration-dependent manner, as determined using flow cytometry, and decreased cell viability and caused plasma membrane damage, as determined using fluorescence microscopy. PP (20 μm, 1 mg/mL) decreased the viability of human dermal fibroblasts (HDFs) by 20% [53]. Similarly, PP (660 ± 270 nm, 4 mg/mL) stimulation could lead to cytotoxicity and death in A549 cells [54]. Through confocal microscopy, Lee et al. reported that 500 nm PS particles were internalized by HUVECs, which induced decreased endothelial cell viability and increased necrotic cell death and LDH release [55]. Although it is not clear whether 500 nm PP particles can be internalized, they also reduced cell viability (350 μg/mL) and increased cell apoptosis, as confirmed using flow cytometry (Figure 2 and Figure 3). Moreover, 300 nm PS could cause the accumulation of autophagosomes in nude mouse leukemia mononuclear macrophages (RAW264.7), and 100 nm PS resulted in the accumulation of autophagosomes in human umbilical vein endothelial cells [37,56]. Surprisingly, 300 nm and 500 nm PP did not significantly promote autophagosome accumulation (Figures S1 and S2), meaning that PP did not enter the cells at these doses, which may be related to the hydrophobicity of PP [57]. Notably, for 500 nm PP, even at a low concentration of 17.5 μg/mL, may be bound to the cytomembrane, subsequently causing cytomembrane damage characterized by significantly increased LDH release (Figure 4B). Compared with previous results, a 3- or 10-fold reduction in PP concentration causing a decrease in cell viability was observed in our data, indicating that the toxicological effects of PP nanoparticles are closely dependent on the particle size and cell type. Moreover, the PP concentration responsible for the significant increase in LDH release was 6-fold greater than that of the PS concentration [55], probably due to the hydrophobicity of PP. In conclusion, the toxicological effects of 500 nm PP-NPs on normal HUVECs cannot be ignored.

Vascular endothelial growth factor (VEGF) stimulates the growth of endothelial cells, and VEGF is essential for the formation of new blood vessels (angiogenesis) and the sprouting and growth of capillaries (angiogenesis) [58]. Endothelial cell migration and angiogenesis are closely related, and endothelial membrane damage promotes endothelial cell differentiation [59]. Angiogenesis is a complex biological process that involves regulating of multiple signaling pathways [60]. Among the downstream signaling pathways, the VEGFA-MAPK signaling pathway is often recognized as an important pathway that promotes vascular endothelial cell proliferation, migration, and angiogenesis [61]. In this study, 500-nm PP-NPs inhibited HUVEC migration, possibly by blocking the signaling pathways downstream of VEGF-MAPK (Figure 5 and Figure 6). Due to the strong hydrophobicity of PP nanoparticles, the concentration that inhibited HUVEC migration was slightly greater than that of PS (40 μg/mL) [55].

Research on the toxicological effects of nanoparticles in terrestrial animals, particularly humans, is still in its infancy. Inflammation and oxidative stress are the most extensively studied mechanisms of cytotoxicity. PP (20 μm, 1 mg/mL) could significantly increase ROS levels by 30% in PBMCs [53]. PP (660 ± 270 nm, 4 mg/mL) stimulation has been found to induce increased ROS levels in A549 cells [54]. PE-NPs (100 nm, 100 μg/mL) can adhere to keratinocyte cells (HaCaT) in a short time and then internalize into cells, leading to decreased cell viability, growth, and proliferation through oxidative stress [62]. In this study, a significant increase in ROS level was induced by 500 nm PP-NPs, even at 1.5 μg/mL (Figure 4Aa,Ab), much lower than those of the other cell types and large-diameter granules described above. Inflammatory factors can induce genetic mutations, leading to cytotoxicity through signal transduction, and eventually cancer in severe cases [63]. In this study, the protein expression of inflammatory cytokines (*IL-6* and *TNF-α*) increased significantly following HUVEC exposure to 500 nm PP-NPs at concentrations of 1.5, 17.5, and 35 μg/mL for 72 h (Figure 7), which is in accordance with their mRNA levels (Figure 8Aa,Ac). The endotoxin contents in the solutions containing 0.1 and 1 mg/mL of 500 nm PP were less than 0.5 EU/mL (Figure S6), helping us to identify the eliminative influence of endotoxin on inflammatory factors; moreover, significant increases in the gene expression of proinflammatory cytokines (*TNF-α*, *IL-2* and *NLRP3*) were observed compared to the control cells (Figure 8Aa–Ad), which were lower than the effective concentrations of 4 mg/mL PP-NPs in A549 cells and 1 mg/mL PP-MPs in PBMCs. Different results concerning NP-induced inflammation and oxidative stress may be related to the characteristics of the cells themselves and the size of NPs. The smaller the NP, the greater the adverse biological effects. In general, cells exposed to extremely high NP concentrations are prone to inflammation and oxidative stress.

Nanoparticles can promote apoptosis by inducing mitochondrial dysfunction and increasing ROS production, ER stress, and DNA damage [64]. Mitochondria are the main source of intracellular ROS, which are released after mitochondrial membrane damage [65]. As the main type of NP particle in plastic-bottle injection [4], PP can directly enter the blood through intravenous injection and flow through various organs, posing a potential threat; however, its effect on the function of normal human cells has rarely been studied. Surprisingly, 48 h exposure to PP-MPs (2–10 μm, 100 μg/mL) has been reported to induce apoptosis in rat H9C2 cells by increasing the expression of *Bax*, *Bcl-2*, and *caspase 3* [66]. It has been found that 24 h of treatment with PS-NPs (50 nm, 150 μg/mL) causes similar changes in the above-mentioned apoptotic factors in human ovarian granulosa tumor cells [67]. Treating of the human hepatoma cell line HepG2 with PET-NPs and PVC-NPs (100 nm, 100 μg/mL) for 24 h achieved a similar proapoptotic effect, as stated above [68]. However, increased HUVEC apoptosis was observed in the nano-PP-exposed group (35 μg/mL) in in vitro experiments. In this study, the expression of mitochondrial apoptosis-related proteins (*caspase-3*, *Bax*, and *p53*) increased significantly, whereas that of *Bcl2* decreased (Figure 8Ba–Bd). P53 induces *Bax* gene expression, translocates to the mitochondrial membrane, activates caspase 3, and initiates apoptosis. *Bcl-2* acts as an antiapoptotic gene, and the ratio of *Bcl-2* to Bax indicates the degree of apoptosis [69]. The concentration of apoptosis-inducing PP-NPs obtained in this study was greater than the above-reported values, possibly related to the cell species and particle type, such as the hydrophobicity of PP [57].

To detect the possible mechanisms leading to cardiovascular disease, RT-PCR analysis was performed to assess changes in relevant factors. Reduced vascular barrier integrity is associated with cardiovascular diseases such as angiogenesis and atherosclerosis [70]. Decreased tight junction protein levels results in barrier dysfunction, potentially inducing increased permeability, cell morphological changes, and decreased proliferation [71]. Vascular barrier dysfunction is considered the initial step in the formation of atherosclerotic plaques [72]. In this study, ZO-1 expression decreased in HUVECs after exposure to PP-NPs (210 μg/mL) (Figure 8C), indicating that cumulative concentrations of NPs introduced from PP-bottled injections may increase the risk of vascular barrier disruption via the depletion of tight junction proteins.

It is known that glucocorticoids (GCs) can inhibit proliferation, migration, and tube formation in HUVECs [73]. Estrogen promotes the proliferation of HUVECs and induces autophagy by inhibiting the Phosphoinositide 3-kinase (PI3K)–RAC-alpha serine/ threonine-protein kinase (AKT)–mammalian target of rapamycin (MTOR)–LC3 signaling pathway [74]. Stress signals can induce apoptosis, oxidative stress, and inflammatory responses in HUVECs cells [75]. In the same manner that PS-NPs activate the ROS-driven NF-κB/NLRP3 pathway, thereby inducing barrier defects in NCM460 cells [76], the main reason for these issues was that increased of TNF-α damaged HUVEC cell membrane, causing the release of LDH, ultimately leading to decreased of ZO-1 levels in this study. However, the decrease in ZO-1 may also be related to the hormone pathways induced by EDCs. Recent studies have shown that RACK1, a key scaffold protein implicated in immune endocrine signaling, also plays a critical role in regulating endothelial junction proteins such as ZO-1 [77] and inflammasomes such as NLRP3 [78]. Furthermore, prior studies have shown that multiple additive-type EDCs can modulate RACK1 in epithelial systems [42]. Due to the hydrophobic nature of PP itself, although it is generally believed that its toxicity is lower than that of PVC and PS, its degradation products and potential additives (such as plasticizers, antioxidants, and catalysts) may have hormonal effects, especially at the nanoscale. Therefore, another possible reason is that the EDCs within PP-NPs may interfere with normal hormone regulation by increasing the inflammatory response, specifically the increase in NLRP3, to downregulate ZO-1 expression, which is similar to the situation where glucocorticoids inducing the downregulation of ZO-1 through RACK1/SRC/E-cadherin, resulting in intestinal damage [78,79]. In summary, at the cellular level, the toxicological damage caused by PP-NPs to HUVECs is mainly characterized by particle-induced oxidative stress and inflammatory responses, with the hormone effects induced by EDCs acting as a secondary component. This is because the latter is more likely to disrupt the balance of glucocorticoids and other substances by interfering with the HPA axis in the body.

In summary, PP-NPs may decrease cell viability through promoting oxidative stress, inflammatory responses, apoptosis, and cytomembrane damage. In comparison, as shown in Table 1, we found that the toxic effects caused by PP nanoparticles and other types of nanoplastics are similar, but we first reported a decrease in the expression of ZO-1 in normal human cells. Therefore, we speculated that the differences in toxicity mainly depend on the type of cells; however, it is worth noting that the signaling pathways that cause the same response may be different. For instance, PP, PE and PS induce the signaling pathways of the NOD-like receptor, JNK-MAPK, and P38-MAPK, sequentially, with all ultimately leading to an inflammatory response [80].

### The Possible Metabolism of MNP Particles In Vivo

As the body has mechanisms for removing particles and thresholds for disease, particle concentrations that are harmful to normal human cells and organoids are not necessarily harmful to humans. First, we discuss the clearance mechanism in the body. Some MNPs inhaled through the nose or ingested through the mouth are removed during their entry into the blood circulation, while others are removed during blood circulation, with only slight amounts being transferred to tissues [81,82]. For example, after 168 h of transbronchial injection with PS, only a small amount (1 μm, 4%; 20 nm, 30%) accumulated in the lung tissues of mice; indeed, 0.5%, 0.2%, and 0.1% of 20-nm particles remained in the kidneys, liver, and spleen during blood circulation, and the clearance rate was nearly 70% [25]. After 28 days of the oral administration of PS-NPs, the percentages of 50/100 nm particles accumulated in the intestine, spleen, and kidneys were 24%, 3%, 3% and 11%, 5%, 0%, respectively, with a clearance rate of 70% at least [83]. Larger particles (>0.2 µm) in the bloodstream are mostly filtered through the spleen or liver and returned to the intestine to be excreted in feces [84], whereas very small particles (<10 nm) can penetrate further into the kidney and are eventually removed via urine; nevertheless, intermediate-sized NPs circulate in the blood and eventually accumulate in the liver, kidneys, heart, reproductive system, and brain [85]. The particles that enter the blood are encapsulated by proteins to form a “protein crown”, which promotes the uptake and internalization of particles by tissue cells, and the size of the particles entering the cells increases to 3 µm [86]. However, some inert plastic particles form protein crowns or are directly degraded by enzymes and become hydrophilic, allowing them to be excreted in urine or faeces (such as PET degradation) [87]. MNPs that accumulate in the blood can cross the cell membrane barrier and initiate an inflammatory response [88] or be cleared from the cell after triggering defense mechanisms [89]. Since the ratio of NP to MP content in infusions used for disease treatment is 100:1 [4], NPs are the only particles that reach the blood through nasal inhalation and oral ingestion [81,84]; therefore, NPs account for the vast majority of particles in human vascular tissue, accounting for approximately 30–90% of such particle accumulation [23]. Moreover, we discuss the MNPs threshold for disease. MNPs (>0.02 μm, 76 μg/g) have been detected in normal human carotid artery tissues, whereas higher amounts (133 μg/g) have been detected in the carotid plaque tissues of patients with atherosclerosis; the latter level may represent the threshold for the disease [90]. In addition, compared with that of the normal population, the risk of nonfatal myocardial infarction, nonfatal stroke, or death from any cause was 4.53 times greater when the plastic particle concentration in carotid plaques reached 13.5 μg/mg [91].

In summary, since the cell experiment was conducted in a relatively closed environment, it could not remove foreign bodies and repair damage, as occurs in the human body; therefore, the experimental concentration may be reduced by the clearance mechanism, which may not reach the pathogenic threshold. In other words, it is possible that more years (>10 years) of particle accumulation through infusion therapy would be required to reach the risk threshold for the risk of vascular injury, though the exact number of years may only found in future in vivo toxicology studies. Currently, there is no need to panic too much, but reducing the use of plastic-bottle injections for infusion would be beneficial.

## 5. Conclusions

In this study, we performed a cell-based toxicological experiment on nanoplastic particles introduced via plastic-bottle injections, using commercial polypropylene nanoparticles to mimic the nanoplastics in injections. In the culture medium containing 0.01% Tween 80, 500 nm polypropylene nanoparticles reduced cell viability, caused cell membrane damage by inducing oxidative stress, inhibited cell migration and wound tissue healing, increased the levels of the inflammatory factors TNF-α and IL-6 (protein and transcription levels), and induced apoptosis by increasing the levels of the apoptotic factors *Bax* and *caspase-3*. Based solely on the changes in the mRNA levels of *NLRP3*, *p53* and *ZO-1*, we preliminarily confirmed that PP-NPs induced inflammation, apoptosis and disruption of the barrier function in HUVECs. In the future, WB experiments could be conducted to further rigorously verify the above-mentioned toxicological effects. This study demonstrated that a certain degree of plastic nanoparticles accumulation could cause damage to HUVECs, which could compensate for the defects caused by polypropylene nanoparticles on other normal cells in the cardiovascular field, in addition to peripheral blood mononuclear cells. Based on a spherical particle used in this study, although they can only being able to indirectly reflect the cellular damage caused by nanoplastics in plastic-bottle injections, this study may provide insights at the cellular level for people who have been treated for diseases using plastic-bottle injections over a long time period. We hope to efficiently transfer plastic fragments previously collected and quantified from plastic-bottle injections and use them for future toxicology studies, especially in vivo studies.

## Figures and Tables

**Table 1:** The toxicological effects on human normal tissue cells and organoids induced by MNPs at concentrations lower than the water environment (40 μg/mL in water).

Cell or Organoid Types	Characteristics of Particles			Toxicological Effects	Ref.
	Type	Size (*Φ*, nm)	Conc. (μg/mL)		
CCD-18Co	PS	500	5	Basal metabolic rewiring, cancerization	[28]
L02	PS	80	12.5	Mitochondrial damage, metabolic disorders	[30]
HEK293	PS	1000	5	Decrease proliferation, glucose metabolism, and antioxidant stress ability	[31]
BMMSCs & AMSCs	PET	<1000	10	Decrease in the number of proliferating cells, and loss of the ability to differentiate	[32]
hiPSC (Forebrain cortex spheroids)	PS	1000 10,000	5	Neuroroxicity	[34]
hiPSC (Intestinal organs)	PS	50	10	Apoptosis and inflammation	[35]
Embryonic stem cells (Hepatoid organs)	PS	1000	0.25	Metabolic dysfunction and lipid accumulation	[36]

hiPSC: human-induced pluripotent stem cell; Conc.: Concentration; Ref: References.

**Table 2:** The sequences of primers used in RT-qPCR analyses.

Gene	Forward (5′ to 3′)	Reverse (5′ to 3′)
*GAPDH*	CTCTGACTTCAACAGCGACA	AAATGAGCTTGACAAAGTGG
*β-actin*	CCCTGGAGAAGAGCTACGAG	TCCATGCCCAGGAAGGAAG
*TNF-α*	TGTTGTAGCAAACCCTCAAG	TTGAAGAGGACCTGGGAG
*Bcl-2*	TTGTGGCCTTCTTTGAGTTC	TTATCCTGGATCCAGGTGTG
*ZO-1*	ACATACATTCTAAGGGAGC	CTCGGTTTGGTGGTCTG
*Caspase-3*	GACTCTGGAATATTCCCTGGACAACA	AGGTTTGCTGCATCGACATCTG
*p53*	AGAGCTGAATGAGGCCTTGGAA	GAGTCAGGCCCTTCTGTCTTGAAC
*Bax*	CATGGGCTGGACATTGGACT	AAAGTAGGAGAGGAGGCCGT
*IL-2*	GTTCTCCTTGCCTAGTGTGGATGG	CCAACAGAGATAACCACGGCTTCC
*IL-6*	GCGCCTTCGGTCCAGTTG	CTCCTTTCTCAGGGCTGAG
*NLRP3*	GGACCTCAGTGACAATTCTC	ACAATCTCCGAATGTTACAG

## Data Availability

The original contributions presented in this study are included in the article/Supplementary Materials. Further inquiries can be directed to the corresponding author.

## Supplementary Materials

The following supporting information can be downloaded at: https://www.mdpi.com/article/10.3390/toxics13090802/s1, Figure S1: Representative micrographs of intracellular localization of LC3B determined by immunofluorescent assay (300 nm); Figure S2: Representative micrographs of intracellular localization of LC3B determined by immunofluorescent assay (500 nm); Figure S3: 500 nm PP-NPs induced inflammatory responses, apoptosis, and a decrease in intercellular junction function in HUVECs; Figure S4: Normal distribution graphs for the data of Figure 2A, Figure 3F, Figure 4Ab,Bf, Figure 5F, Figure 6H, Figure 7A–D and Figure 8; Figure S5: The particle sizes of PP 500 nm determination by Nanoparticle size and potential analyzer (NS90Z, Malvern Panalytical); Figure S6: The result of endotoxin determination by Limulus amebocyte lysate test. A. The standard curve of endotoxin reference material. B. Endotoxin determination results of the control and experimental samples (Dilute the PP-NPs solution at a concentration of 10 mg/mL with endotoxin test water). Table S1: Zeta potential of samples.
