# Supplementary material for: Toxicological Impacts of Polypropylene Nanoparticles Similar in Size to Nanoplastics in Plastic-Bottle Injections on Human Umbilical Vein Endothelial Cells
Source: Toxics. 2025 Sep 21;13(9):802. doi: 10.3390/toxics13090802 (PMC12474203; doi:10.3390/toxics13090802)
Supplement: Supplementary file 1 [file toxics-13-00802-s001.zip › toxics-3810268-supplementary.pdf]

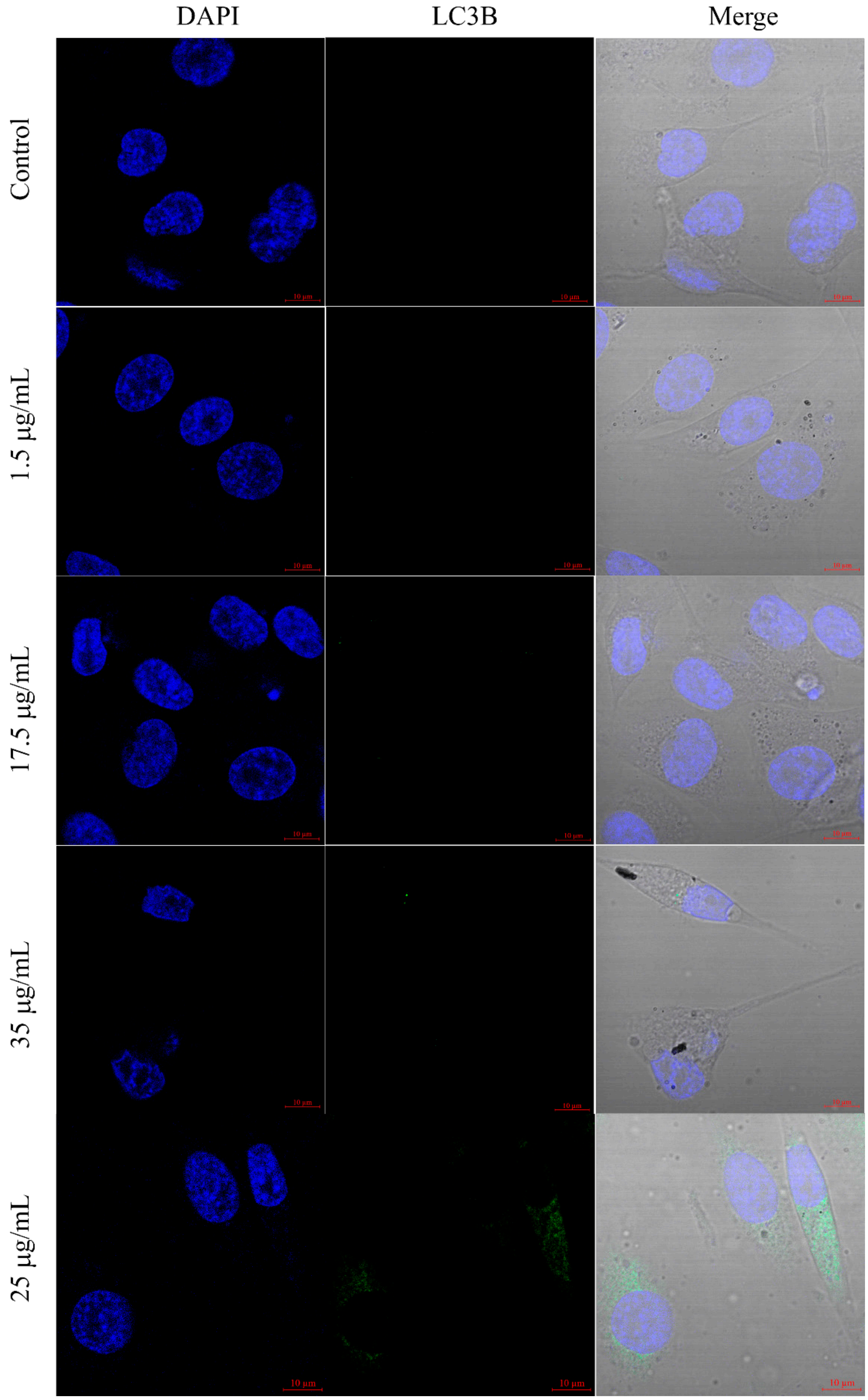


**Figure S1.** Representative micrographs of intracellular localization of LC3B determined by immunofluorescent assay. Nucleus was stained with DAPI (blue), LC3B was probed with a primary anti-LC3B (green). Cells were exposed to 1.5, 17.5, 35 μg/mL of 300 nm PP-NPs and 25 μg/mL of 100 nm PP-NPs for 48 h, respectively.

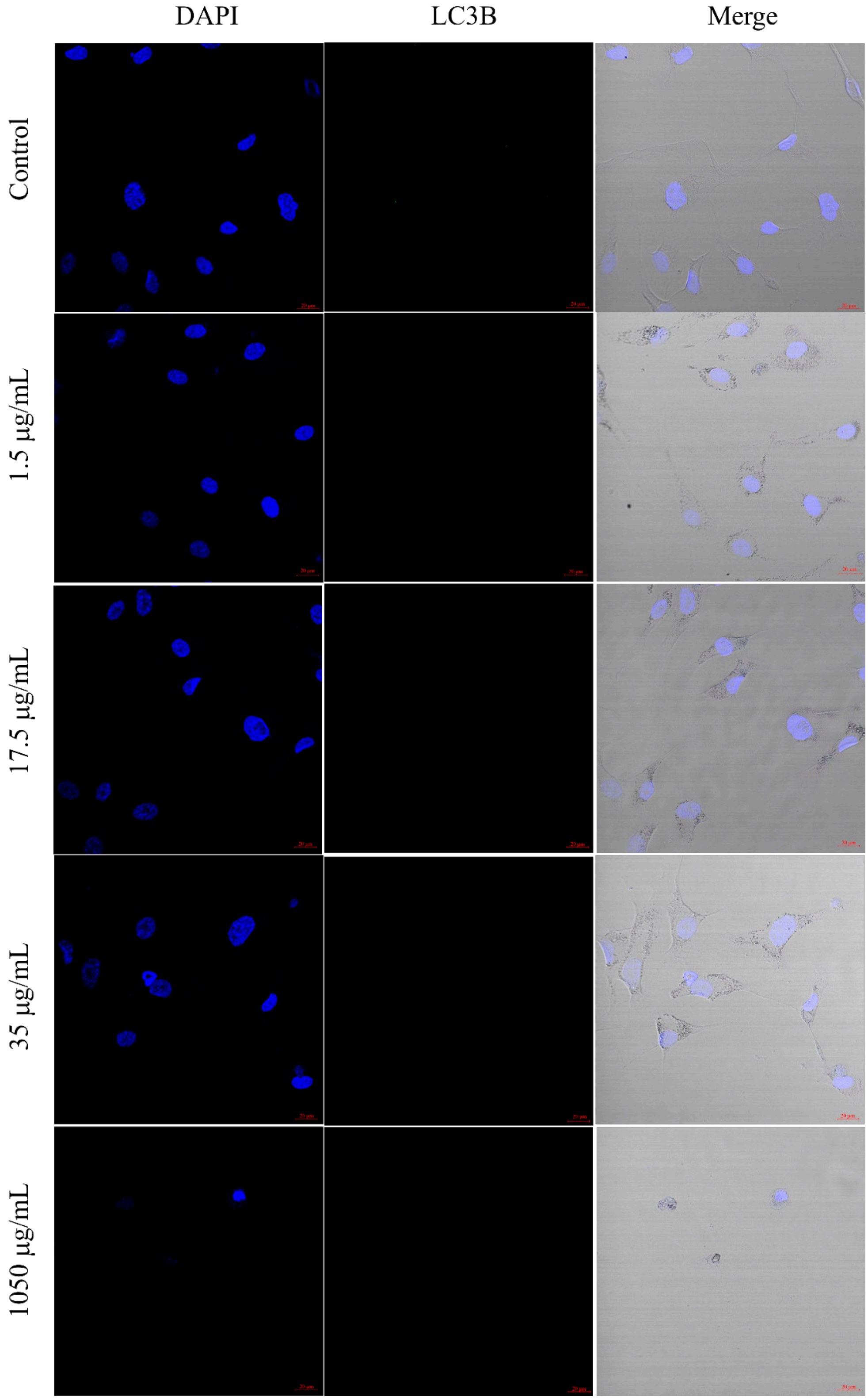


**Figure S2.** Representative micrographs of intracellular localization of LC3B determined by immunofluorescent assay. Nucleus was stained with DAPI (blue), LC3B was probed with a primary anti-LC3B (green). Cells were exposed to 1.5, 17.5, 35.0, 1050 μg/mL of 500 nm PP-NPs for 48 h, respectively.

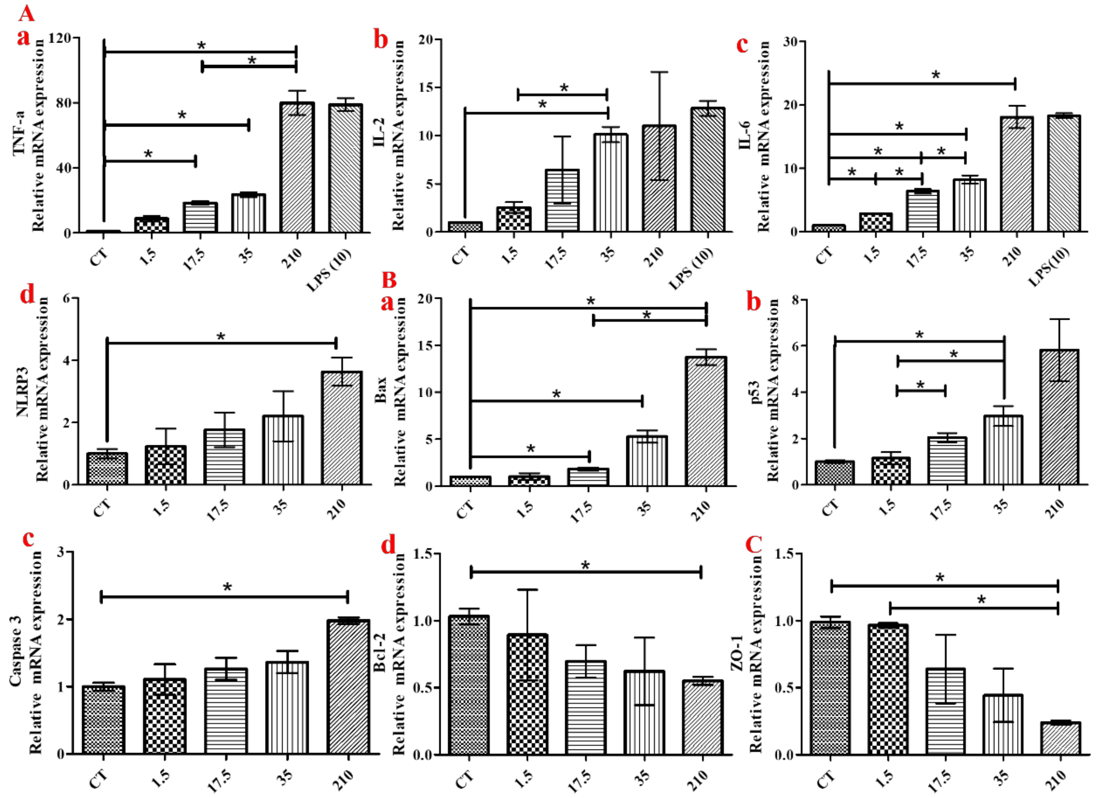


**Figure S3.** 500 nm PP-NPs induced inflammatory responses, apoptosis, and a decrease in intercellular junction function in HUVECs. (Aa-Ad, Ba-Bd, C) The mRNA levels of *TNF-α* (Aa), *IL2* (Ab), *IL6* (Ac), *NLRP3* (Ad), *Bax* (Ba), *p53* (Bb), *Caspase-3* (Bc), *Bcl-2* (Bd) and *ZO-1* (C). Cells were incubated with different concentrations (0, 1.5, 17.5, 35 and 210 μg/mL) of PP-NPs for 48 h, respectively. The data were mean ± SEM (n = 5). The mRNA levels of above factors were normalized to *β-actin.* *$p < 0.05$.

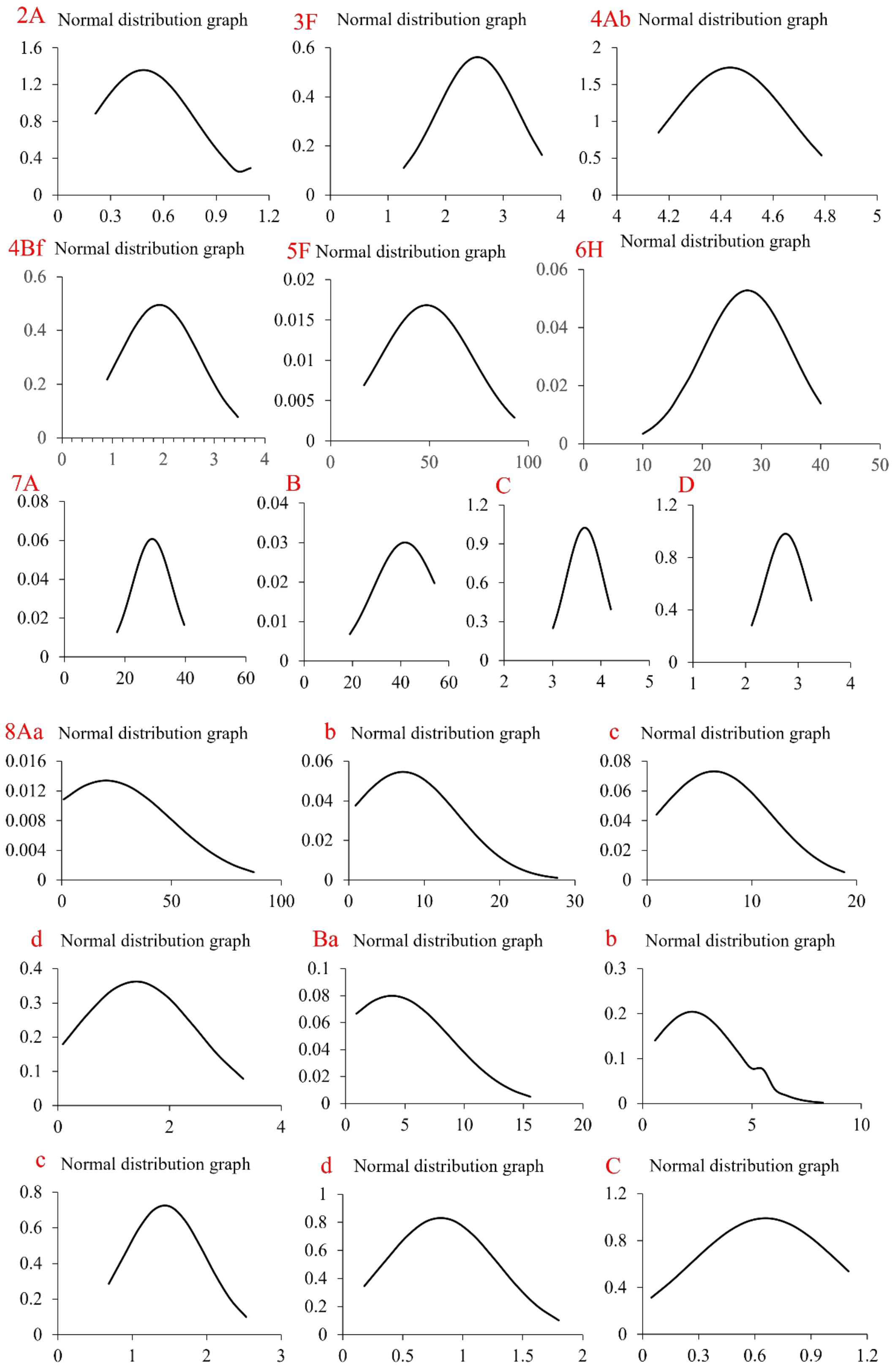


**Figure S4.** Normal distribution graphs for the data of Figures 2A, 3F, 4Ab, 4Bf, 5F, 6H, 7A-D, 8.

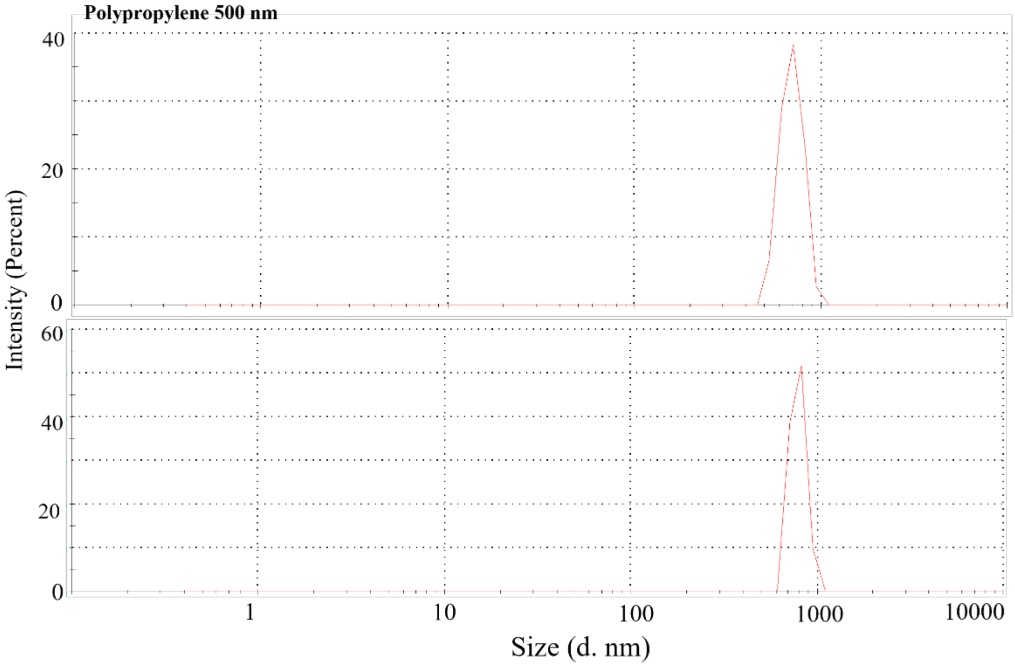


**Figure S5.** The particle sizes of PP 500 nm determination by Nanoparticle size and potential analyzer (NS90Z, Malvern Panalytical).

A

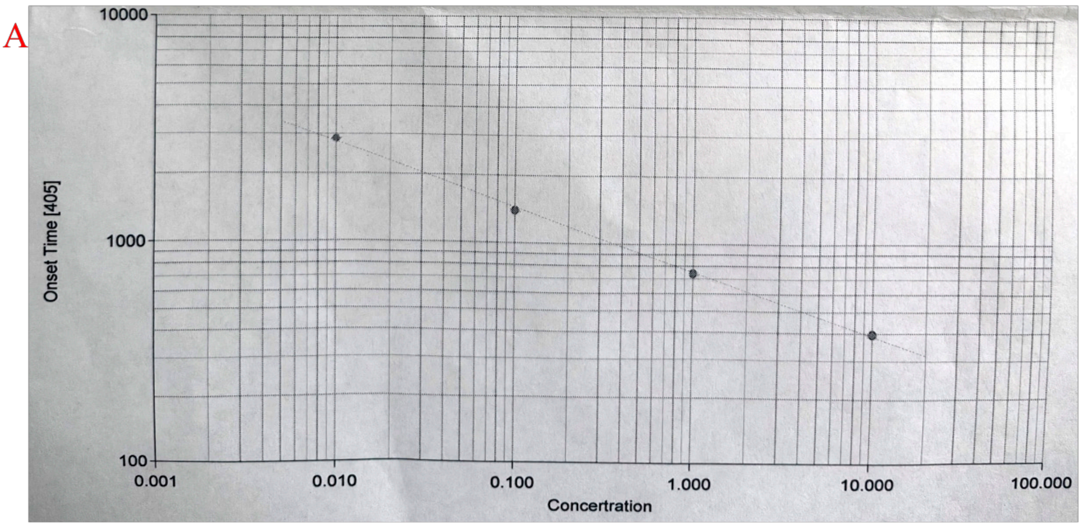


B

$$\mathrm{Log}(Y)=A*\mathrm{Log}(X)+B \qquad R^2=0.999$$

| **Name** | **Well** | **Dilution** | **Concentration** | **Concentration ×Dilution** | **Average (EU/mL)** |
|---|---|---|---|---|---|
| 0.01%Tween<br>80+medium | B4<br>B5 | 1<br>1 | <0.005<br><0.005 | <0.005<br><0.005 | <0.005 |
| 0.01%Tween<br>80+medium | D4<br>D5 | 10<br>10 | <0.005<br><0.005 | <0.05<br><0.05 | <0.05 |
| 1 mg/mL PP-NPs<br>(0.01%Tween 80<br>+medium) | B6<br>B7 | 10<br>10 | <0.005<br>0.012 | <0.05<br>0.12 | <0.085 |
| 0.1 mg/mL PP-NPs<br>(0.01%Tween<br>80+medium) | D6<br>D7 | 100<br>100 | <0.005<br><0.005 | <0.5<br><0.5 | <0.5 |

**Figure S6.** The result of endotoxin determination by Limulus amebocyte lysate test. A. The standard curve of endotoxin reference material. B. Endotoxin determination results of the control and experimental samples (Dilute the PP-NPs solution at a concentration of 10 mg/mL with endotoxin test water).

**Table S1.** Zeta potential of samples.

| Samples | Measurement of Zeta potential (mV) | | | |
|---|---|---|---|---|
| | $1_{st}$ | $2_{nd}$ | $3_{rd}$ | Average |
| Physiological saline solution containing only NPs - 0.01% Tween 80 (Nanoplastics 6.6 μg/L) | -2.38 | -3.55 | -4.32 | -3.41 |
| Physiological saline solution without MNPs-0.01% Tween 80 (PP 500 nm 6.6 μg/L) | -2.67 | -2.84 | -3.94 | -3.15 |

The detection was performed by Laser nanoparticle size analyzer (NS90, Omec, Zhuhai, China). Sample 1: The filtrate after filtering out microplastics with GF-B membrane ($\Phi$= 1 μm) was then added with 0.01% Tween 80 to obtain sample 1; Sample 2: The filtrate after filtering out micro and nano plastics with an $Al_2O_3$ membrane was then successively added with 0.01% Tween 80 and PP nanoparticles to obtain sample 2.
